# Ergot

**Published:** 1842-03

**Authors:** 


*Ergot.*—Dr. Wright inclines to the opinion that the formation of this excrescence is owing to the combined influence of atmospheric heat and moisture. He is confirmed in this opinion by ergot being much more common in the district of Soloyre—a moist, rich soil, hot and sheltered—than elsewhere. Grains also occur, only one half of which is ergotted, an insuperable objection to the notion that ergot is produced by the growth of a fungus, which would of course attack all parts of the grain. Besides, from his own experiments, he finds that ergot in powder or grains, sowed with rye, fails to produce the disease in the growing plant. Watering the growing plants freely, with water in which ergot had been steeped, failed in like manner; so did the application of ergot to the growing ears of rye. The excresence remarked at the upper extremity of the grain, he regards as the *stigmata* altered by disease.

Dr. Wright's conclusions were further confirmed by discovering that ergot contains a considerable quantity (26 per cent.) of fecula, which it would not do if the disease was produced by fungus. Dr. Wright has the merit of discovering fecula in ergot—no previous analyst makes mention of it.—*N. Y. Med. Gaz. from Ed. Med. & Surg. Jour.*

